# Ship Classification and Anomaly Detection Based on Spaceborne AIS Data Considering Behavior Characteristics

**DOI:** 10.3390/s22207713

**Published:** 2022-10-11

**Authors:** Zhenguo Yan, Xin Song, Hanyang Zhong, Lei Yang, Yitao Wang

**Affiliations:** College of Aerospace Science and Engineering, National University of Defense Technology, Changsha 410073, China

**Keywords:** spaceborne AIS, ship behavior characteristics, ship classification, anomaly detection, maritime surveillance

## Abstract

With the establishment of satellite constellations equipped with ship automatic identification system (AIS) receivers, the amount of AIS data is continuously increasing, and AIS data have become an important part of ocean big data. To further improve the ability to use AIS data for maritime surveillance, it is necessary to explore a ship classification and anomaly detection method suitable for spaceborne AIS data. Therefore, this paper proposes a ship classification and anomaly detection method based on machine learning that considers ship behavior characteristics for spaceborne AIS data. In view of the characteristics of different types of ships, this paper introduces the extraction and analysis of ship behavior characteristics in addition to traditional geometric features and discusses the ability of the proposed method for ship classification and anomaly detection. The experimental results show that the classification accuracy of the five types of ships can reach 92.70%, and the system can achieve better results in the other classification evaluation metrics by considering the ship behavior characteristics. In addition, this method can accurately detect anomalous ships, which further proves the effectiveness and feasibility of the proposed method.

## 1. Introduction

As the most effective long-distance transportation mode at present, maritime transportation accounts for more than 90% of the world’s trade volume [1]. With the rapid growth in the number of ships, the maritime traffic environment is becoming increasingly complex, maritime traffic accidents occur occasionally, and the pressure on maritime traffic monitoring is increasing [2]. With the continuous development of information and communication technology, to better implement maritime traffic management so as to further reduce the collision probability, the ship automatic identification system (AIS) emerges at a historic moment. Since 2004, the International Maritime Organization (IMO) has made it mandatory for international sailing ships with 300 gross tonnage and upwards and all passenger ships to be equipped with AIS equipment [3], and the spatial and temporal AIS data of ships have increased rapidly. AIS data have been widely used in maritime traffic characteristic discovery, ship behavior analysis and anomaly detection, maritime safety supervision, maritime situational awareness, maritime rescue, ocean pollution, and ship monitoring [4]. In addition, traditional maritime navigation and route planning mostly rely on charts, radar, and other information, and are then judged by the crew’s long-term navigation experience, which leads to a low degree of automation and the predominance of human factors. Research shows that 80% to 85% of recorded maritime accidents are directly caused by or related to human error [5], while the introduction of the AIS system is expected to further improve the automation level of offshore operations and the quality of channel management.

The AIS is a tracking and self-reporting system that can identify and locate ships through communication among nearby ships and base stations [6], and provides additional accurate information about the target ships. AIS data can provide rich ship information, including static information (e.g., ship name, size, and type), dynamic information (e.g., ship position, speed, and heading), and voyage-related information (e.g., ship draught and destination), which can be recorded by shore-based stations or space-based AIS receivers [7]. However, because shore-based AIS cannot fully and seamlessly cover the entire ocean space, the use of AIS installed on satellites makes it possible to monitor ships around the world and further expand the application scope of AIS data. Compared with other types of ocean data, such as remote sensing images, AIS data involve the near-real-time collection and recording of global ship navigation information, which provides a large amount of research data for studying the spatial and temporal characteristics of maritime supervision and ship behavior patterns.

At present, although a large amount of AIS data can be obtained, the interpretation speed of AIS data processing methods is far less than that of AIS data reception and cannot fully mine the information behind it, which means that it is highly necessary to develop an efficient and intelligent method to cope with the great deal of information. In recent years, the continuous development and innovation of artificial intelligence technologies, such as machine learning and deep learning methods, have provided advanced algorithm tools for humans to mine and use the knowledge behind AIS data. The rapid accumulation of spaceborne AIS data provides massive information for maritime traffic control and ship behavior analysis, and the use of data mining technology to mine the potential maritime traffic characteristics of AIS data to realize maritime surveillance has become a research hotspot. Examples include ship trajectory reconstruction and prediction [8,9,10,11,12], ship collision avoidance [13,14,15,16], ship behavior analysis and anomaly detection [1,17,18,19], global oil trade analysis, and maritime environment monitoring [7,20].

It can be found that the current AIS data mining works have penetrated all aspects of maritime traffic and ship behavior research, which also proves the strong advantage of AIS data compared with other ship data sources. In recent years, with the development of machine learning and deep learning methods, an increasing number of scholars have applied these methods to AIS data analysis to realize in-depth data mining. These methods can overcome the problems wherein the previous statistical analysis of AIS data required relevant professional knowledge background and precise assumptions to a certain extent, greatly promoting the intellectualization of maritime traffic management and ship navigation based on AIS data and further deepening the understanding of maritime ship behavior. Nonetheless, the application of AIS data in maritime surveillance still faces many difficulties.

In recent years, the development of AIS has promoted research on ship behavior patterns, and the description of maritime traffic characteristics has been more detailed than in previous studies. AIS data have been successfully applied to ship motion analysis, ship collision risk assessment, and ship classification and anomaly detection, which has effectively promoted the development of maritime surveillance technology. However, some ships with AIS evade detection and perform illegal operations or even illegal exploration by turning off the transponder, forging position data, and deliberately transmitting false identity information [21]. In addition, AIS data also have problems such as mislabeling of ship type information, data imbalance, and poor data quality [22,23]. These problems hinder the further mining of AIS data and pose many challenges to maritime surveillance.

As the basis of AIS data mining, ship classification can be used to determine the types of ships that play a crucial role in promoting the application of maritime surveillance. At present, some existing research works have achieved a certain effect by extracting ship features and combining them with machine learning methods (e.g., SVM, KNN, and CNN) for ship classification. However, most of these studies have some disadvantages, such as the few ship types classified, incomplete ship feature extraction, and short time and small range of AIS data used. In addition, the performance of ship classification methods has yet to be improved. If more global dynamic information of AIS data can be considered, it will be beneficial to obtain more comprehensive ship behavior characteristics for ship monitoring. This paper proposes a ship classification and anomaly detection method based on spaceborne AIS data for ships worldwide by considering ship behavior characteristics. Based on the AIS data collected by ocean series satellites, this paper makes full use of the advantages of spaceborne AIS data, such as wide coverage, long tracking time, and rich ship types, and combines machine learning methods to conduct AIS data mining to improve the monitoring ability of ships in the open sea. This paper focuses on spaceborne AIS data mining in maritime surveillance, namely ship classification and anomaly detection. The major contributions of this paper are the following:

(i) In the process of ship classification and identification, we carried out a comprehensive feature extraction project on the global AIS data to form 13-dimensional features, including geometric features and behavior characteristics (course distance and sailing speed were considered), which enriches the input of classification models.
(ii) We conducted research on the ship classification of spaceborne AIS data based on machine learning algorithms. The influence of different classifiers and feature combinations on the classification performance was analyzed and discussed. Experiments showed that the performance of classifiers can be improved by using the extracted behavior characteristics.
(iii) Case studies of ship anomaly detection were presented and analyzed, and the experimental results demonstrate that the proposed method can effectively solve the ship anomaly detection problem in maritime surveillance.

The remainder of this paper is organized as follows: Section 2 reviews ship classification methods based on AIS data. Section 3 introduces the implementation of the proposed method in detail. This section first analyzes the spaceborne AIS data, including data preprocessing and ship type analysis. Then, aiming at the feature engineering analysis of classification problems, this section extracts the geometric features and behavioral characteristics of ships. Section 4 presents the experimental results and analysis, including the classification results of the proposed method and case studies of ship anomaly detection. Finally, the conclusions of this paper and future research directions are discussed in Section 5.

## 2. Related Work

At present, research on ship classification and anomaly detection based on data mining technology and massive AIS information has made great progress. Ship classification mainly involves ship feature extraction and feature-based ship type identification. Different types of ships have unique appearances and behavior patterns. For example, cargo ships are usually large- and medium-sized ships carrying out ocean transportation tasks, while fishing ships are generally small- and medium-sized ships engaged in offshore activities [24]. Compared with other types of maritime data, AIS data contain rich static, motion, and ship tag information, making them more suitable for ship classification. In this section, studies related to AIS data ship classification are reviewed.

Pedroche et al. [25] proposed a trajectory-based AIS data fishing vessel classification architecture, which was processed by dividing ship trajectories into fishing vessels and non-fishing vessels as a binary classification. Damastuti et al. [26] used the K-NN algorithm to classify ships sailing in Indonesian waters, and tested the algorithm on a real-time AIS database with a time range of 3–4 months. Sheng et al. [27] proposed three basic modes of ship motion according to the characteristics of ship motion and designed a classification algorithm for correlation sub-trajectories. Based on the features extracted from ship trajectories, a logistic regression model was used to build ship classifiers to solve the classification problems of fishing and cargo ships. Elwakdy et al. [28] divided each ship trajectory into multiple segments and extracted each sub-trajectory as input features using a polynomial function. Subsequently, a ship classifier based on an adaptive neuropathy fuzzy inference system (ANFIS) was constructed to classify oil tankers and fishing ships. Zhong et al. [29] extracted the geometric features of ships based on the sizes of ships (i.e., ship length and width) in spaceborne AIS messages and used the random forest algorithm to classify three types of ships, i.e., cargo ships, oil tankers, and fishing ships. Zhou et al. [30] proposed a regional ship behavior clustering method by analyzing the AIS data of the Rotterdam port and classified ships into corresponding behavior clusters according to the static characteristics of ships. Kraus et al. [31] considered the German Bight as the research object and conducted ship classification research on three-month AIS data collected by shore-based and spaceborne AIS receivers. Wang et al. [32] extracted the static information of ships in AIS data and used random forest to classify ships.

Ship anomaly detection is of great significance for maritime safety and intelligent supervision. In recent years, ship classification based on AIS data has been applied to anomaly detection. For example, Zhen et al. [18] used hierarchical clustering combined with k-medoids clustering to learn typical sailing patterns and then used a Naïve Bayes classifier to find anomalous ships. Handayani et al. [33] used a support vector machine (SVM) to classify and identify two types of anomalies: U-turn and anomalous parking routes. Kira Kowalska et al. [34] used the Gaussian process to learn the normal sailing modes of ships from AIS data for anomaly detection and used an activation learning paradigm to select formatted sub-samples to reduce the complexity of training. Mazzarella et al. [35] proposed a Bayesian method called the knowledge-based particle filter (KB-PF) for ship position prediction, which requires prior use of the K-nearest neighbor (KNN) algorithm to classify ship routes. Nguyen et al. [36] proposed a multi-task deep learning architecture based on variational recurrent neural networks (VRNNs). This method re-encodes AIS data similarly to “one-hot” and inputs the AIS data stream into a deep neural network for training so that it can complete multiple tasks, such as trajectory reconstruction, anomaly detection, and ship type classification and identification.

It can be observed that current ship classification studies based on AIS data mainly include two aspects: ship type identification and anomaly analysis. The former focuses on the classification of ship types by extracting ship static and motion features. The latter can detect the anomalous motion state mainly by analyzing the sailing state of ships. From the above research status, it can be seen that ship classification research based on AIS data still has the following problems:

(i) At present, the AIS data used for ship classification are mostly collected by shore-based AIS stations; therefore, the ship motion mode is relatively singular. This is because the coverage of shore-based AIS stations is relatively small. They can only monitor the maritime traffic situation in a specific sea area, which has certain limitations. Compared with shore-based AIS stations, spaceborne AIS receivers can realize AIS data collection worldwide and carry out large-scale offshore data mining. However, there are relatively few studies on ship behavior analysis and maritime traffic control in the open sea for spaceborne AIS.
(ii) Most current studies on ship classification are focused on cargo ships and oil tankers, which account for the vast majority of ships, resulting in a relatively singular type of ship classification for AIS data. The development of maritime surveillance technology requires research on various types of ships, but relatively few studies have been conducted on passenger ships, fishing ships, and other types of ships.
(iii) The existing studies mainly focus on the single geometric features of ships, and few studies consider the ship behavior characteristics, which is the necessary direction to further improve the performance of AIS ship classification.

In general, current research on ship classification based on AIS data mainly focuses on a single ship feature mode in a small geographical range and timespan, and a comprehensive analysis of a large range and multiple features is still lacking. Shore-based AIS data can only reflect the spatiotemporal motion information of ships in a very small range, whereas space-based AIS can realize long-term and large-scale data reception, which is more suitable for ship classification and anomaly detection. Since the concept of spaceborne AIS has been proposed, especially with the construction of AIS constellations and the development of commercial operations, the quality of spaceborne AIS data has steadily improved. In the public literature, relatively few studies have been performed for spaceborne AIS data analysis. Making use of the advantages of spaceborne AIS data to perform more effective data mining is an important direction for ocean big data analysis and the intelligent development of maritime surveillance in the future. Therefore, this paper considers spaceborne AIS data as the research object and extracts the geometric features and behavior characteristics of ships to realize ship classification and anomaly detection.

## 3. Materials and Methods

With the increasing application of AIS, a large amount of ship data has been provided for maritime surveillance. The interval of a ship broadcasting AIS messages is determined by the information type and navigation status. The reporting interval of static information is 6 min, and the reporting interval of dynamic information is between 2 s and 3 min [37]. This means that the development of high-performance ship classification and identification algorithms to handle such massive information is challenging. This paper comprehensively extracts ship features and studies the ship classification and anomaly detection method for spaceborne AIS data based on machine learning methods. The experimental results show that the proposed method can effectively improve the performance of spaceborne AIS data mining and the application levels of maritime surveillance by considering ship motion features.

### 3.1. AIS Data Source

**Figure 1:**
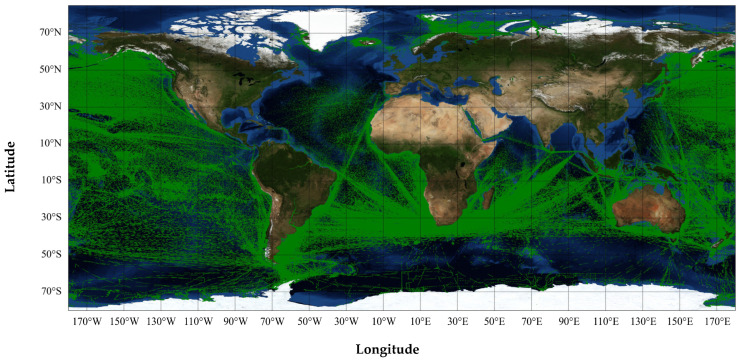
The partial AIS data received by the HY-1C satellite.

To meet the needs of monitoring global ships, the Haiyang-1C (HY-1C) and Haiyang-2B (HY-2B) satellites are all equipped with highly sensitive AIS receivers. The monitoring widths of the HY-1C and HY-2B satellites are better than 950 km and 1000 km, respectively. The HY-1C and HY-2B satellites are in sun-synchronous orbits (SSO), and can achieve the ability to collect information and monitor global ocean ships twice a day. These two satellites can obtain the location and attribute information of global maritime ships, and provide data services for the maintenance of maritime rights and interests, marine disaster prevention and mitigation, and marine fishery production activities. Based on the AIS data received by the HY-1C and HY-2B satellites, this paper makes full use of the advantages of the wide coverage and rich ship navigation information of the spaceborne AIS data to perform ship classification and anomaly detection research. Figure 1 shows a chart drawn from the partial AIS data received by the HY-1C satellite.

This paper analyzes the AIS data received by the HY-1C and HY-2B satellites within one year, and mainly studies messages 1, 2, 3, and 5 among 27 types of AIS messages. These messages comprehensively cover the static and dynamic information of ships, including ship maritime mobile service identification (MMSI), size, type, position, draught, rate of turn (*ROT*), speed over ground (*SOG*), and heading information. The AIS data used in this paper are shown in Table 1.

### 3.2. AIS Data Preparation and Analysis

#### 3.2.1. Data Preprocessing

Due to satellite signal transmission and human factors, the AIS messages received by satellites may have problems such as data error and incomplete format that are not conducive to analysis in practical applications [5]. To address the adverse effects of these issues, data preprocessing such as data cleaning and calibration is required. Data preprocessing can provide reliable AIS data for subsequent ship classification and anomaly detection. AIS data preprocessing mainly consists of format error screening and data deduplication. Format error screening aims to delete the noise data in messages 1, 2, 3, and 5 that do not conform to AIS format specifications and omit important information. Duplicate data are defined as messages with the same MMSI and time. According to the statistics of the AIS database, such messages usually have the same longitude and latitude information, so only one duplicate AIS message is reserved. In addition, there is a very small number of “duplicate data” with different longitude and latitude information, which cannot be judged as correct or incorrect, so it will not be considered in this paper. Furthermore, the “type” attribute field for messages 1, 2, and 3 is created and filled according to the “ship and cargo type” attribute field of message 5. By preprocessing the AIS data, 62 million messages for various types of ships were obtained.

#### 3.2.2. Ship Type Statistics and Analysis

**Figure 2:**
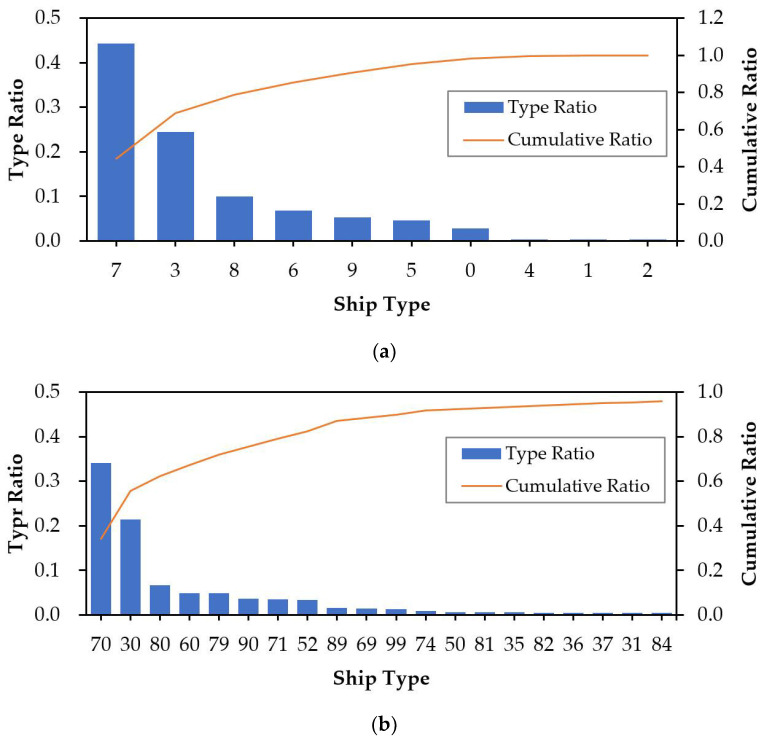
The distribution of ship types in the AIS data. (**a**) The distribution of ship types by ship category codes; (**b**) the distribution of the top 20 ship types with the largest number by ship subcategory code.

Because different types of ships have different behavior characteristics, it is necessary to conduct ship type analysis to determine whether the types of classified ships are representative. Therefore, this paper adopts the database self-matching method to statistically analyze the AIS data. The AIS message 5 contains static information such as ship types. Different types of ships have unique type codes [37]. The first and second digits of ship type codes represent the ship type and supplementary information, respectively. Figure 2 shows the distribution of the AIS data ship types.

According to the definition of the “ship and cargo type” attribute field of message 5, AIS data define each type of ship as a unique code [37]. The codes of passenger ships, cargo ships, tanker ships, fishing ships, and tug ships are “60–69”, “70–79”, “80–89”, “30”, and “52”, as shown in Table 2. The ship category code “9” refers to other types of ships, and code “0” has no specific type definition. These two types of ships are not considered in spaceborne AIS data analysis. Figure 2a,b show the distribution of ship types by ship category code and the top 20 ship types with the largest number of messages by ship subcategory code. It can be observed that cargo ships, tanker ships, fishing ships, passenger ships, and tug ships are the top five types of ships with the largest proportion. These ships are representative ships sailing at sea and can provide massive sample data for training classification models.

### 3.3. Ship Feature Extraction

Feature extraction is a key step in ship classification and anomaly detection. Whether the selected features can reflect the differences between different ship types largely determines the classification performance. AIS data contain the ship’s length, width, speed, position, heading, and other information. In this paper, ship features were divided into geometric features and behavior characteristics, which were analyzed and extracted. Based on these two types of features, machine learning algorithms were used to classify and identify five types of important ships sailing in the ocean, i.e., cargo ships, tanker ships, fishing ships, passenger ships, and tug ships.

#### 3.3.1. Geometric Feature Extraction

**Figure 3:**
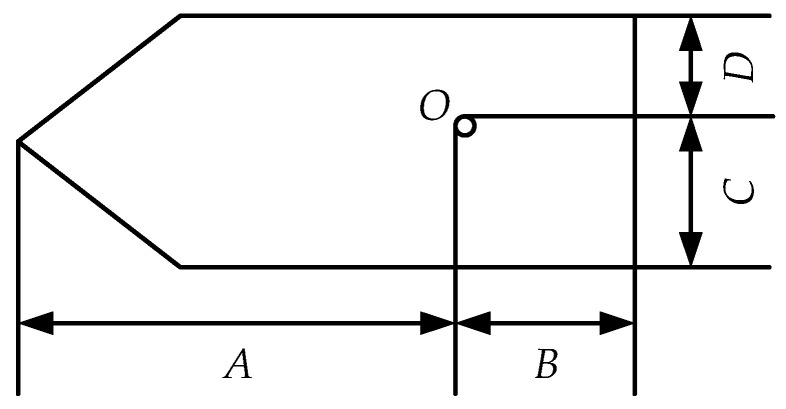
The reference point for reporting position and overall dimensions of a ship.

Static information message 5 of AIS data contains the length and width information of the ship, and its fields *A*, *B*, *C*, and *D* reflect the overall dimensions of a ship, which, respectively, represent the distances from reference point *O* for reporting position to the bow, stern, port side, and starboard, as shown in Figure 3.

Then, the features *Length* and *Width* can be calculated by Equation (1):

(1) $$\left\{ \begin{array}{l} Length=A+B\\ Width=C+D \end{array}\right.$$

In general, the measurement of ship appearance characteristics also includes *Naive_Perimeter*, *Naive_Area*, *Aspect_Ratio*, and *Shape_Complex* [38]. The relevant definitions are given in Equation (2):

(2) $$\left\{ \begin{array}{l} Naive\_Perimeter=2\times (Length+Width)\\ Naive\_Area=Length\times Width\\ Aspect\_Ratio=Length/Width\\ Shape\_Complex={\left(Length+Width\right)}^{2}/(Length\times Width) \end{array}\right.$$

Finally, the 6-dimensional feature shown in Equation (3) was selected as the geometric feature input for the ship classification algorithm.

(3) $$\begin{array}{ll} f_{g}= & [Length,\;Width,\;Naive\_Perimeter,\;Naive\_Area,\\ & Aspect\_Ratio,\;Shape\_Complex] \end{array}$$

Table 3 shows the statistical results of the mean value and standard deviation (Std. deviation) of the geometric features for the five selected types of ships. It can be observed that different types of ships exhibit certain differences in the extracted features. The *Length* and *Width* of ocean sailing cargo ships and oil tankers are significantly larger than those of the other three types of ships, and their geometric features are relatively similar. By contrast, fishing ships, passenger ships, and tug ships are generally small offshore ships. As can be seen from the statistical results, fishing ships and passenger ships are similar in the *Aspect_Ratio* and *Shape_Complex*, and the *Naive_Area* and *Naive_Perimeter* of fishing ships and tug ships are similar; however, the size of passenger ships is slightly larger than that of fishing ships and tug ships.

#### 3.3.2. Behavior Characteristic Extraction

To improve the accuracy of ship classification, this paper adds to the extraction of behavior characteristics besides geometric features. The behavioral descriptions of ships are more complex. Cargos and tankers often carry out cross-ocean transportation across continents. Their activity range is much larger than that of small ships such as fishing ships, passenger ships, and tug ships, and their speed is relatively stable when sailing in the ocean, whereas fishing ships, passenger ships, and tug ships are flexible and their speed changes quickly. Based on the above analysis and the fact that spaceborne AIS data can achieve the global monitoring of ships, this paper conducts a ship behavior study within the time range of one year. Messages 1, 2, and 3 of the AIS data contain the longitude, latitude, *SOG*, and other dynamic ship information. According to Equation (4), the features *Longitude_Span* (unit: °), *Latitude_Span* (unit: °), and *Voyage_Distance* (unit: km) of each ship can be calculated as the motion trajectory characteristics of ships in this paper. Assuming that the *i*-*th* ship has *m* (*m* > 1) AIS messages, then

(4) $$\left\{ \begin{array}{l} Longitude\_Span=\left|longitude_{\max}-longitude_{\min}\right|\\ Latitude\_Span=\left|latitude_{\max}-latitude_{\min}\right|\\ Voyage\_Distance=\sum_{k=1}^{m-1}2R\cdot \arcsin\left(\sqrt{\sin^{2}(\frac{\Delta \varphi_{k}}{2})+\cos(rlat_{k})\cdot \cos(rlat_{k+1})\cdot \sin^{2}(\frac{\Delta \lambda_{k}}{2})}\right) \end{array}\right.$$

where $\Delta \varphi_{k}=\left|rlat_{k+1}-rlat_{k}\right|$ and $\Delta \lambda_{k}=\left|rlon_{k+1}-rlon_{k}\right|$ represent the absolute values of the latitude and longitude differences between two adjacent points, *rlat* and *rlon* represent the radian values of the corresponding latitude and longitude, and *R* is the average radius of the Earth, which is taken as 6371 km in this paper.

In addition, through a statistical analysis of the speeds of various types of ships in the AIS database, this paper divides the speeds of ships (*SOG*) into high-speed and low-speed sailing states according to whether it is greater than five knots. According to Equation (5), this paper calculates the mean value and standard deviation of the two states to describe the average speed and speed change scale of ships, respectively; thus, four additional ship behavior characteristics (i.e., *High_Speed_Mean*, *High_Speed_Std*, *Low_Speed_Mean*, and *Low_Speed_Std*) can be obtained. Suppose that there are *n* AIS messages with SOG > 5 in *m* AIS messages of ship *i*, then

(5) $$\left\{ \begin{array}{l} High\_Speed\_Mean=\left(\sum_{h=1}^{n}SOG_{h}\right)/n\\ High\_Speed\_Std=\left[\sum_{h=1}^{n}{\left(SOG_{h}-High\_Speed\_Mean\right)}^{2}\right]/n\\ Low\_Speed\_Mean=(\sum_{l=1}^{m-n}SOG_{l})/\left(m-n\right)\\ Low\_Speed\_Std=\left[\sum_{l=1}^{m-n}{\left(SOG_{l}-Low\_Speed\_Mean\right)}^{2}\right]/\left(m-n\right) \end{array}\right.$$

where 0<n<m. In the case of *n* = 0 or *n* = *m*, this paper automatically fills it with the average value of the corresponding features of this type of ship. The extracted 7-dimensional motion features are expressed in Equation (6):

(6) $$\begin{array}{ll} f_{m}= & [Longitude\_Span,\;Latitude\_Span,\;Voyage\_Distance,\\ & High\_Speed\_Mean,\;High\_Speed\_Std,\\ & Low\_Speed\_Mean,\;Low\_Speed\_Std] \end{array}$$

The statistical results of the ship behavior characteristics on the dataset are shown in Table 4. It can be seen that the annual activity range of cargo ships and tanker ships is much larger than that of the other three types of ships. The speed of fishing ships at high speeds is generally lower than that of other ships, but their speed is the highest at low speeds, which reflects the characteristics of the frequent maneuvering of fishing ships in fishing operations. Compared with other types of ships, tugs have a smaller value in *Longitude_Span*, *Latitude_Span*, and *Voyage_Distance*, which is in line with the working characteristics of a small tug operation range and infrequent dispatch. Therefore, the selected motion characteristics can better describe the differences between different ship types.

## 4. Experimental Results and Analysis

To verify the effectiveness of extracted behavior characteristics for AIS data ship classification and anomaly detection, we selected the classical machine learning algorithm support vector machine (SVM) [39] and random forest (RF) [40] as the implementation method. Both the algorithms have been widely used in classification tasks. All experiments in this section were programmed and implemented in the Python 3.8 environment under Windows 10. We spliced the preprocessed AIS messages into long-term ship behavior sequences based on MMSI codes, and 18,200 samples of five types of ships were finally formed through feature extraction, as described in Section 3.3.1. During the experiment, 200 AIS data samples of each type of ship were selected as test samples, and the rest were used as training samples. The AIS data used in this paper were all from the HY-1C and HY-2B satellites.

In this paper, the common classifier evaluation metrics are used to evaluate the proposed method, including *Accuracy*, *Precision*, *Recall*, and *F*_1_*-Score*. The relevant calculation is shown in Equation (7):

(7) $$\begin{array}{l} Accuracy=\frac{TP+TN}{TP+TN+FP+FN}\\ Precision=\frac{TP}{TP+FP}\\ Recall=\frac{TP}{TP+FN}\\ F_{1}-Score=\frac{2\times Precision\times Recall}{Precision+Recall} \end{array}$$

where, *TP*, *FP*, *FN*, *TN* represent True Positives, False Positives, False Negatives, and True Negatives, respectively.

### 4.1. Ship Classification Considering Geometric Features

This section only adopts the geometric features *f_g_* of ships as the input of the SVM and random forest. The classification accuracy of the trained SVM and random forest model on the test dataset is presented in Table 5, and the maximum classification accuracy of the models is marked in bold. It can be observed that when the geometric features are used, the highest classification accuracy of the test dataset is 73.10%. The classification accuracy of the random forest is better than that of the SVM, and the random forest also shows its advantages in other evaluation metrics. In addition, compared with fishing ships, passenger ships, and tug ships, the *F*_1_*-Score* of cargo ships and tanker ships is relatively high. However, the overall performance of both the classifiers is poor, which could be improved by using more ship features.

**Figure 4:**
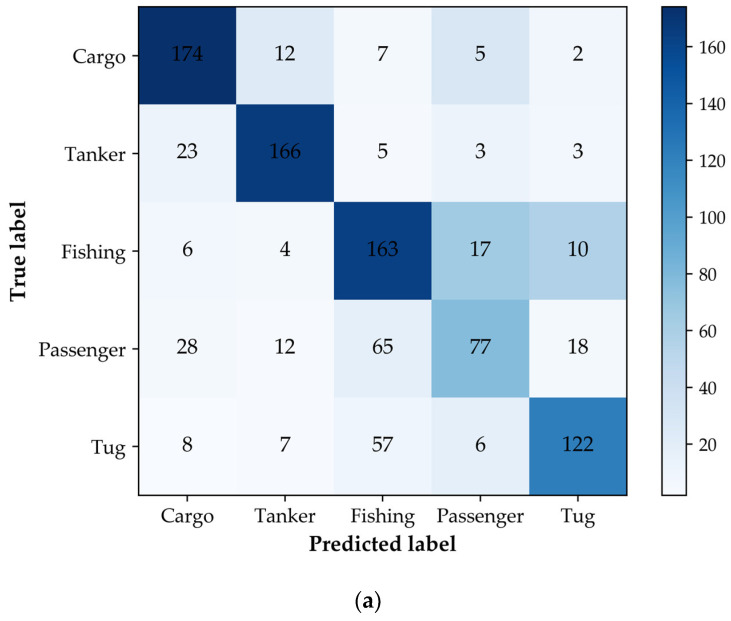

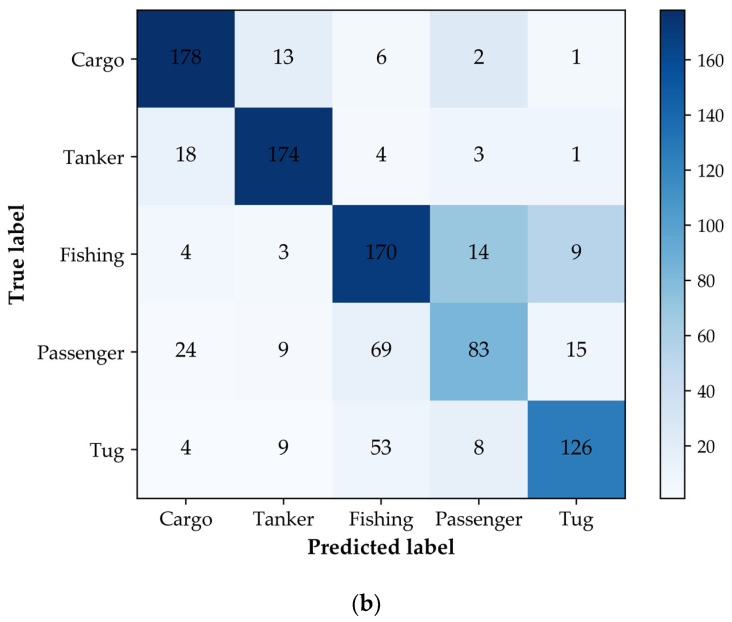
The confusion matrix of the five types of ship test samples considering the geometric features. (**a**) The confusion matrix of the SVM; (**b**) the confusion matrix of the RF.

Figure 4 shows the classification confusion matrixes of the SVM and random forest on the test dataset. The “true label” is the real label of ships in the AIS data, and the “predicted label” is the classification result of the classifiers. It can be seen that there is classification confusion in the prediction results of both the classifiers. The number of misclassification samples of the random forest is less than that of the SVM. It is worth noting that cargo ships and tanker ships are easily confused in the process of classification, which can be explained by the fact that these two types of ships have certain similarities in shape design and construction. Fishing ships, passenger ships, and tug ships also have classification confusion, which is more serious than that of cargo ships and tanker ships. When the geometric features are considered, both the classifiers are prone to misclassifying ship samples. In order to improve the classification confusion, it is necessary to adopt more distinguishing ship features.

### 4.2. Ship Classification Considering Geometric Features and Behavior Characteristics

According to the discussion in the previous section, the classification accuracy of the five types of ships can reach approximately 70% when the geometric features are considered. In this section, the geometric features *f_g_* and behavior characteristics *f_m_* are considered simultaneously—that is, the input of the SVM and the random forest is a 13-dimensional feature vector. The classification accuracy of the classifiers on the test dataset is presented in Table 6, and the maximum classification accuracy is marked in bold. The classification accuracy of the SVM and random forest reached 87.40% and 92.70%, which are significantly higher than those of the models when only the geometric features are considered. In addition, the other evaluation metrics of the classifiers have also been improved by adding behavior characteristics. For example, when the random forest model is used during the experiment, the *F*_1_*-Score* of the five types of ships reaches 92.80%, 92.11%, 92.91%, 93.88%, and 93.20%, respectively. It can also be found that the overall classification result of the RF model is better than that of the SVM, which shows strong performance in the AIS data ship classification tasks. The experiments show that the behavior characteristics constructed in this paper are effective in the task of AIS data ship classification.

**Figure 5:**
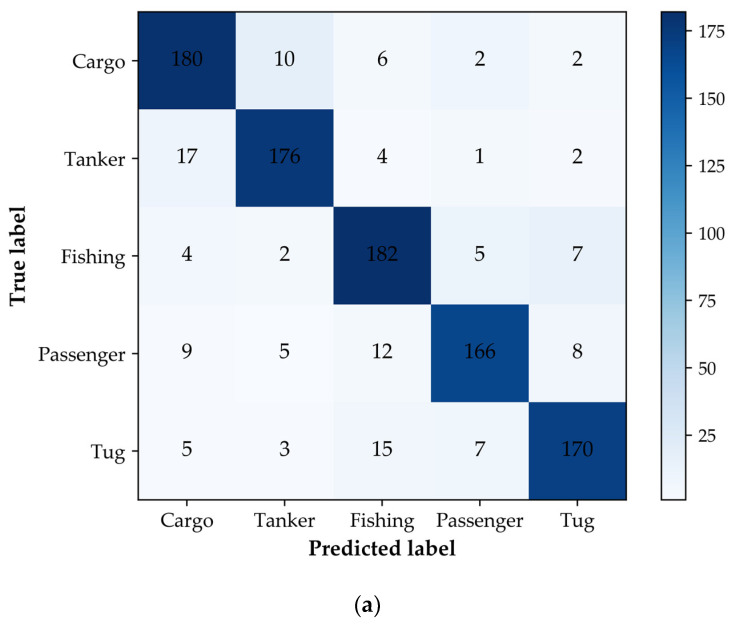

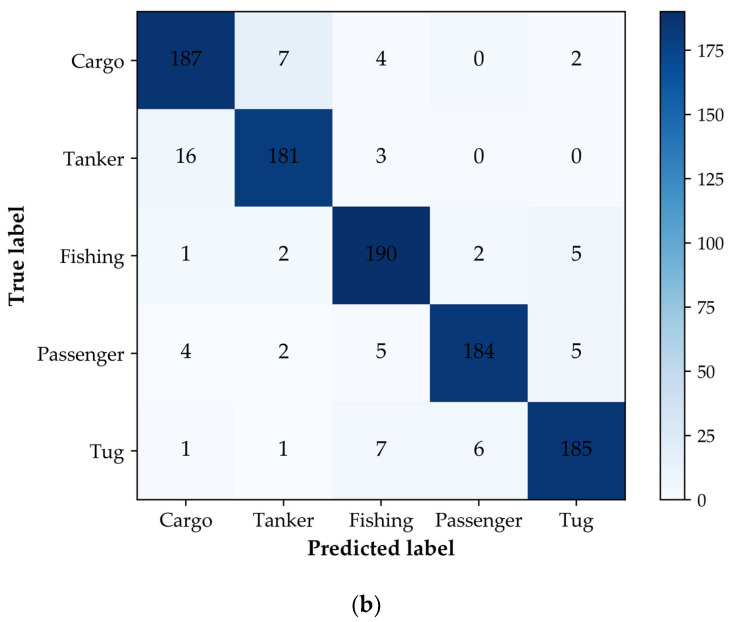
The confusion matrix of the five types of ship test samples considering the geometric features and behavior characteristics. (**a**) The confusion matrix of the SVM; (**b**) the confusion matrix of the RF.

Figure 5 shows the classification confusion matrixes of the five types of ships for the SVM and RF models. Due to the similarity in the spatial distribution of features between cargo ships and tanker ships, as well as fishing ships, passenger ships, and tug ships, the output of the classifiers still has classification confusion. However, comparing Figure 4 and Figure 5, it can be seen that the misclassification of the five types of ships, especially passenger ships and tugs, has been significantly improved by adding behavior characteristics. From the experimental results presented in Section 4.1 and Section 4.2, it can be seen that the performance of the ship classification models is significantly improved when the extracted behavior characteristics are used, which demonstrates the effectiveness of our method.

### 4.3. Analysis of Ship Anomaly Detection Results

From the above experimental analysis, it can be seen that ships with similar shapes and motion patterns are likely to cause classification confusion, such as cargo ships and tanker ships, fishing ships, passenger ships, and tug ships. However, there are also some cases in which the classification labels of some ships are quite different from their message types. For example, in Figure 4 and Figure 5, the labels of ships in AIS messages are cargo ships, whereas the model gives fishing ship labels. This may be because these ships evade detection by forging and transmitting inappropriate AIS messages. As an important part of maritime surveillance, ship anomaly detection is of great significance for ensuring maritime safety. In this section, case studies are conducted on ships with anomalous classification results to verify the effectiveness of the proposed method.

**Figure 6:**
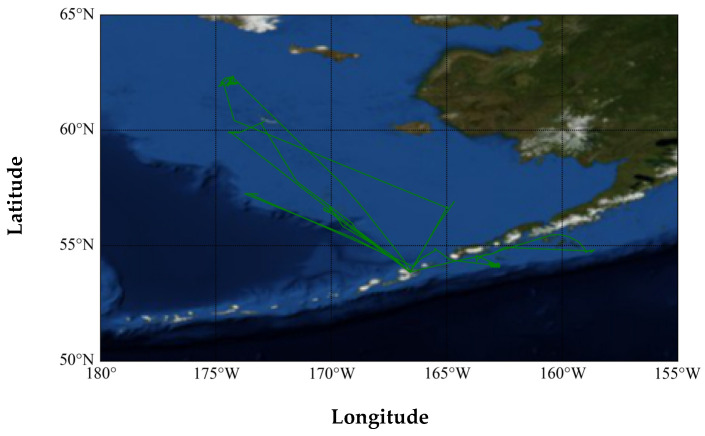
The historical trajectory of the ship (MMSI: 367588710).

In this section, a ship with the MMSI code 367588710 was first selected from the anomalous classification results for anomaly detection analysis. In the AIS database, the type attribute field of its messages shows that it is a cargo ship, whereas the output of the classification model in this paper is a fishing ship. According to the trajectory drawn by its historical AIS messages, as shown in Figure 6, it can be seen that the ship’s movement range is small, the longitude range is less than 5°, and the movement trajectory is complicated, which is inconsistent with the trajectory characteristics of a cargo ship’s linear long-distance navigation.

**Figure 7:**
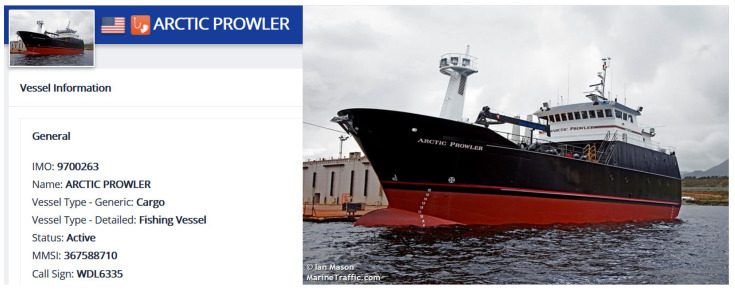
The registration information and photos of the ship (MMSI: 367588710) on the Marine Traffic website.

The Marine Traffic website [41] is one of the most popular online ship tracking service websites in the world. It can provide a real-time chart of global maritime routes online, including ship pictures, ship origin, ship destination, estimated route, IMO number, etc. The registered photos and relevant information about this ship (MMSI: 367588710) on the Marine Traffic website are shown in Figure 7. It can be seen that this ship does not conform to the characteristics of general cargo ships in appearance, as there is a common fishing rod structure among fishing ships. In summary, the ship is likely an anomalous ship that is a fishing ship but broadcasts the wrong ship type.

**Figure 8:**
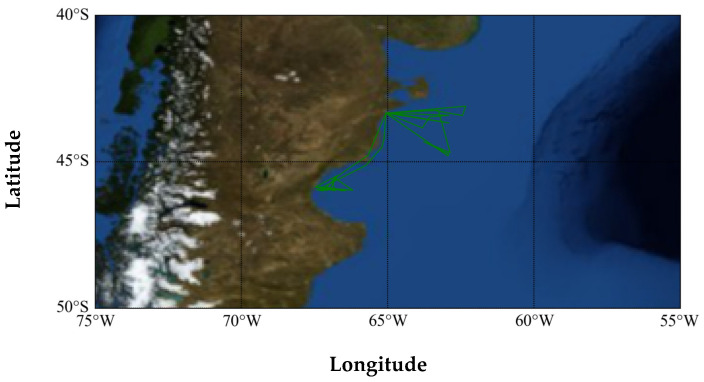
The historical trajectory of the ship (MMSI: 701006130).

In this section, the ship (MMSI: 701006130), whose message attribute is displayed as a passenger ship in the AIS database, is selected for anomaly detection analysis. The classification model proposed in this paper classifies this ship as a fishing ship. Figure 8 shows the historical trajectory of this ship. It can be seen that the motion range of this ship is small, and the range of longitude and latitude does not exceed 5°. In addition, the trajectory of this ship is staggered and complicated, which is inconsistent with the trajectory characteristics of the passenger ship; thus, this ship is also very likely to be an anomalous ship that is a fishing ship but broadcasts the wrong ship type.

**Figure 9:**
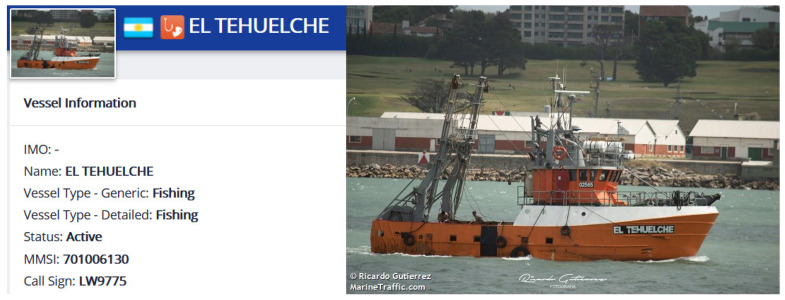
The registration information and photos of the ship (MMSI: 701006130) on the Marine Traffic website.

The registration information and photos of this ship retrieved from the Marine Traffic website are shown in Figure 9. It can be seen that the type of this ship is a fishing ship, which further proves the effectiveness of the proposed method in this paper.

## 5. Conclusions and Future Work

To promote the application of spaceborne AIS data in maritime surveillance, this paper proposed a ship classification and anomaly detection method considering ship behavior characteristics. The proposed method makes full use of the advantages of the wide coverage of spaceborne AIS data to extract distinctive behavior characteristics and combined machine learning algorithms to achieve high-performance classification of the five types of ships (i.e., cargo ships, tanker ships, fishing ships, passenger ships, and tug ships). This paper first analyzed and extracted the geometric features and behavior characteristics of ships, and then used the SVM and random forest algorithms to conduct classification and anomaly detection research. Experiments were performed with different classification models and feature combinations. Through a summary of the experimental results, it was found that the classification accuracy of the five types of ships could reach 92.70% with the addition of ship behavior characteristics, and the method achieved better performance in the *Precision*, *Recall*, and *F*_1_*-score* metrics. In addition, this paper also presented case studies of ship anomaly detection, and the experimental results showed that the proposed method can effectively detect anomalous ships that may forge and transmit inappropriate AIS messages in order to evade monitoring. This is of great significance to ensure maritime economic security and environmental stability, and also proves the effectiveness of the method proposed in this paper.

In the future, research works will focus on ship feature extraction and classifier design. The performance of AIS data ship classification could also be improved by extracting additional features, such as ship draught, *ROT*, and *COG* in AIS messages. The fusion of AIS data and remote sensing images for ship classification is also a future research direction, which could further promote the application of AIS data in maritime surveillance.

## Figures and Tables

**Table 1:** Introduction to the AIS data used.

Satellites	Message Types	Fields Selected
HY-1C/HY-2B	Message 1 (dynamic information)	MMIS, Time, Time Stamp, Longitude, Latitude, SOG, A, B, C, D, Type
	Message 2 (dynamic information)	
	Message 3 (dynamic information)	
	Message 5 (Static information)	

**Table 2:** The corresponding relationships between AIS ship type codes and ship types.

Ship Subcategory Codes	Ship Types	Ship Category Codes
30	Fishing ship	3
52	Tug ship	5
60–69	Passenger ship	6
70–79	Cargo ship	7
80–89	Tanker ship	8

**Table 3:** The statistical results of ship geometric features.

Geometric Features	Evaluating Indicators	Ship Types				
		Cargo	Tanker	Fishing	Passenger	Tug
*Length* (*m*)	Mean value	212.64	215.96	50.88	97.75	43.31
	Std. deviation	66.66	70.70	24.82	82.16	37.68
*Width* (*m*)	Mean value	32.73	36.65	10.36	16.89	12.56
	Std. deviation	9.58	12.97	5.61	10.76	6.86
*Naive_Perimeter* (*m*)	Mean value	490.75	505.21	122.49	229.23	111.74
	Std. deviation	150.94	166.49	57.35	183.40	87.25
*Naive_Area* (*m*^2^)	Mean value	7540.11	8795.10	614.26	2420.80	761.77
	Std. deviation	4228.87	5555.35	914.04	3293.65	1814.57
*Aspect_Ratio*	Mean value	6.48	5.97	4.94	5.25	3.29
	Std. deviation	0.94	0.64	1.36	1.78	1.09
*Shape_Complex*	Mean value	8.64	8.14	7.17	7.47	5.63
	Std. deviation	0.92	0.63	1.27	1.69	1.01

**Table 4:** The statistical results of ship behavior characteristics.

Motion Features	Evaluating Indicators	Ship Types				
		Cargo	Tanker	Fishing	Passenger	Tug
*Longitude_Span* (°)	Mean value	200.21	166.74	86.49	69.56	22.16
	Std. deviation	135.13	130.17	126.8	120.54	58.08
*Latitude_Span* (°)	Mean value	60.01	53.95	21.16	29.99	10.42
	Std. deviation	27.93	26.51	24.07	43.91	18.07
*Voyage_Distance* (*km*)	Mean value	65,373.68	54,165.88	33,637.14	33,934.47	9440.48
	Std. deviation	39,046.90	71,069.34	144,369.14	45,860.41	20,906.18
*High_Speed_Mean* (*knot*)	Mean value	12.42	12.35	10.49	13.49	9.02
	Std. deviation	3.42	3.18	10.49	8.64	8.52
*High_Speed_Std* (*knot*)	Mean value	1.93	1.69	2.77	2.88	2.55
	Std. deviation	2.10	1.74	5.64	3.95	5.65
*Low_Speed_Mean* (*knot*)	Mean value	1.53	1.52	2.04	1.21	1.37
	Std. deviation	0.95	0.91	1.02	0.80	0.89
*Low_Speed_Std* (*knot*)	Mean value	1.15	1.07	1.07	1.13	1.19
	Std. deviation	0.52	0.51	0.37	0.48	0.43

**Table 5:** The classification results of the five types of ships considering the geometric features.

Methods	Ship Types	Evaluation Metrics				
		*Precision*	*Recall*	*F*_1_*-Score*	Support Size	*Accuracy*
SVM	Cargo	72.80%	87.00%	79.27%	200	70.20%
	Tanker	82.59%	83.00%	82.79%	200	
	Fishing	54.88%	81.50%	65.59%	200	
	Passenger	71.30%	38.50%	50.00%	200	
	Tug	78.71%	61.00%	68.73%	200	
RF	Cargo	78.07%	89.00%	83.18%	200	**73.10%**
	Tanker	83.65%	87.00%	85.29%	200	
	Fishing	56.29%	85.00%	67.73%	200	
	Passenger	75.45%	41.50%	53.55%	200	
	Tug	82.89%	63.00%	71.59%	200	

**Table 6:** The classification results of the five types of ships by considering both the geometric features and behavior characteristics.

Methods	Ship Types	Evaluation Metrics				
		*Precision*	*Recall*	*F*_1_*-Score*	Support Size	*Accuracy*
SVM	Cargo	83.72%	90.00%	86.75%	200	87.40%
	Tanker	89.80%	88.00%	88.89%	200	
	Fishing	83.11%	91.00%	86.88%	200	
	Passenger	91.71%	83.00%	87.14%	200	
	Tug	89.95%	85.00%	87.40%	200	
RF	Cargo	92.12%	93.50%	92.80%	200	**92.70%**
	Tanker	93.78%	90.50%	92.11%	200	
	Fishing	90.91%	95.00%	92.91%	200	
	Passenger	95.83%	92.00%	93.88%	200	
	Tug	93.91%	92.50%	93.20%	200	

## Data Availability

Not applicable.

## References

[1] Pallotta G., Vespe M., Bryan K. (2013). Vessel pattern knowledge discovery from AIS data: A framework for anomaly detection and route prediction. Entropy.

[2] Chuah L.F., Mokhtar K., Bakar A.A., Othman M.R., Osman N.H., Bokhari A., Mubashir M., Abdullah M.A., Hasan M. (2022). Marine environment and maritime safety assessment using port state control database. Chemosphere.

[3] Pan S., Jingbo Y. (2018). Extracting shipping route patterns by trajectory clustering model based on automatic identification system data. Sustainability.

[4] Fournier M., Casey Hilliard R., Rezaee S., Pelot R. (2018). Past, present, and future of the satellite-based automatic identification system: Areas of applications (2004–2016). WMU J. Marit. Aff..

[5] Harati-Mokhtari A., Wall A., Brooks P., Jin W. (2007). Automatic Identification System (AIS): Data reliability and human error implications. J. Navig..

[6] Wei Z., Xie X., Zhang X. (2020). AIS trajectory simplification algorithm considering ship behaviours. Ocean. Eng..

[7] Yan Z., Xiao Y., Cheng L., Chen S., Zhou X., Ruan X., Li M., He R., Ran B. (2020). Analysis of global marine oil trade based on automatic identification system (AIS) data. J. Transp. Geogr..

[8] Sang L.Z., Wall A., Mao Z., Yan X.P., Wang J. (2015). A novel method for restoring the trajectory of the inland waterway ship by using AIS data. Ocean. Eng..

[9] Murray B., Perera L.P. (2020). A dual linear autoencoder approach for vessel trajectory prediction using historical AIS data. Ocean. Eng..

[10] Liu J., Shi G., Zhu K. (2019). Vessel trajectory prediction model based on AIS sensor data and adaptive chaos differential evolution support vector regression (ACDE-SVR). Appl. Sci..

[11] Gao D.W., Zhu Y.S., Zhang J.F., He Y.K., Yan K., Yan B.R. (2021). A novel MP-LSTM method for ship trajectory prediction based on AIS data. Ocean. Eng..

[12] Liu R.W., Liang M., Nie J., Lim W.Y.B., Zhang Y., Guizani M. (2022). Deep learning-powered vessel trajectory prediction for improving smart traffic services in maritime internet of things. IEEE Trans. Netw. Sci. Eng..

[13] Zhang W., Goerlandt F., Montewka J., Kujala P. (2015). A method for detecting possible near miss ship collisions from AIS data. Ocean. Eng..

[14] Bakdi A., Glad I.K., Vanem E., Engelhardtsen Y. (2019). AIS-based multiple vessel collision and grounding risk identification based on adaptive safety domain. J. Mar. Sci. Eng..

[15] Nguyen M.C., Zhang S., Wang X. (2018). A novel method for risk assessment and simulation of collision avoidance for vessels based on AIS. Algorithms.

[16] Hörteborn A., Ringsberg J.W. (2021). A method for risk analysis of ship collisions with stationary infrastructure using AIS data and a ship manoeuvring simulator. Ocean. Eng..

[17] Kroodsma D.A., Mayorga J., Hochberg T., Miller N.A., Boerder K., Ferretti F., Wilson A., Bergman B., White T.D., Block B.A. (2018). Tracking the global footprint of fisheries. Science.

[18] Zhen R., Jin Y., Hu Q., Shao Z., Nikitakos N. (2017). Maritime anomaly detection within coastal waters based on vessel trajectory clustering and Naïve Bayes Classifier. J. Navig..

[19] Rong H., Teixeira A.P., Soares C.G. (2020). Data mining approach to shipping route characterization and anomaly detection based on AIS data. Ocean. Eng..

[20] Liu B., Zhang W. (2021). Tracing illegal oil discharges from vessels using SAR and AIS in Bohai Sea of China. Ocean. Coast. Manag..

[21] Mccauley D.J., Woods P., Sullivan B., Bergman B., Jablonicky C., Roan A., Hirshfield M., Boerder K., Worm B. (2016). Ending hide and seek at sea. Science.

[22] Iphar C., Napoli A., Ray C. Detection of false AIS messages for the improvement of maritime situational awareness. Proceedings of the Oceans 2015-MTS/IEEE Washington.

[23] Liang M., Zhan Y., Liu R.W. (2021). MVFFNet: Multi-view feature fusion network for imbalanced ship classification. Pattern Recognit. Lett..

[24] Chuah L.F., Mohd Salleh N.H., Osnin N.A., Alcaide J.I., Abdul Majid M.H., Abdullah A.A., Bokhari A., Jalil E.E.A., Klemeš J.J. (2021). Profiling Malaysian ship registration and seafarers for streamlining future Malaysian shipping governance. Aust. J. Marit. Ocean. Aff..

[25] Pedroche D.S., Amigo D., García J., Molina J.M. (2020). Architecture for trajectory-based fishing ship classification with AIS data. Sensors.

[26] Damastuti N., Aisjah A.S., Masroeri A.A. Classification of ship-based automatic identification systems using k-nearest neighbors. Proceedings of the 2019 International Seminar on Application for Technology of Information and Communication (iSemantic).

[27] Sheng K., Liu Z., Zhou D., He A., Feng C. (2018). Research on ship classification based on trajectory features. J. Navig..

[28] Elwakdy M., El-Bendary M., Eltokhy M. A Novel Trajectories Classification Approach for different types of ships using a Polynomial Function and ANFIS. Proceedings of the 2015 International Conference on Image Processing, Computer Vision, & Pattern Recognition (IPCV’15).

[29] Zhong H., Song X., Yang L. Vessel classification from space-based ais data using random forest. Proceedings of the 2019 5th International Conference on Big Data and Information Analytics (BigDIA).

[30] Zhou Y., Daamen W., Vellinga T., Hoogendoorn S.P. (2019). Ship classification based on ship behavior clustering from AIS data. Ocean. Eng..

[31] Kraus P., Mohrdieck C., Schwenker F. Ship classification based on trajectory data with machine-learning methods. Proceedings of the 2018 19th International Radar Symposium (IRS).

[32] Wang Y., Yang L., Song X., Li X. (2021). Ship classification based on random forest using static information from AIS data. Journal of Physics: Conference Series, Proceedings of the 2021 4th International Conference on Mechatronics and Computer Technology Engineering (MCTE 2021).

[33] Handayani D., Sediono W., Shah A. Anomaly detection in vessel tracking using support vector machines (SVMs). Proceedings of the 2013 International Conference on Advanced Computer Science Applications and Technologies.

[34] Kowalska K., Peel L. Maritime anomaly detection using Gaussian process active learning. Proceedings of the 2012 15th International Conference on Information Fusion.

[35] Mazzarella F., Arguedas V.F., Vespe M. Knowledge-based vessel position prediction using historical AIS data. Proceedings of the 2015 Sensor Data Fusion: Trends, Solutions, Applications (SDF).

[36] Nguyen D., Vadaine R., Hajduch G., Garello R., Fablet R. A multi-task deep learning architecture for maritime surveillance using AIS data streams. Proceedings of the 2018 IEEE 5th International Conference on Data Science and Advanced Analytics (DSAA).

[37] Union I.T. Technical Characteristics for an Automatic Identification System Using Time Division Multiple Access in the VHF Maritime Mobile Band. http://www.itu.int/rec/R-REC-M.1371/en.

[38] Lang H., Wu S., Xu Y. (2018). Ship classification in SAR images improved by AIS knowledge transfer. IEEE Geosci. Remote Sens. Lett..

[39] Cortes C., Vapnik V.N. (1995). Support vector networks. Mach. Learn..

[40] Breiman L. (2001). Random forests. Mach. Learn..

[41] Traffic M. The Marine Traffic Website. http://www.marinetraffic.com.

